# A Random Sequential Mechanism of Aminoglycoside Acetylation by *Mycobacterium tuberculosis* Eis Protein

**DOI:** 10.1371/journal.pone.0092370

**Published:** 2014-04-03

**Authors:** Oleg V. Tsodikov, Keith D. Green, Sylvie Garneau-Tsodikova

**Affiliations:** Department of Pharmaceutical Sciences, College of Pharmacy, University of Kentucky, BioPharm Complex, Lexington, Kentucky, United States of America; University of Padova, Medical School, Italy

## Abstract

An important cause of bacterial resistance to aminoglycoside antibiotics is the enzymatic acetylation of their amino groups by acetyltransferases, which abolishes their binding to and inhibition of the bacterial ribosome. Enhanced intracellular survival (Eis) protein from *Mycobacterium tuberculosis* (*Mt*) is one of such acetyltransferases, whose upregulation was recently established as a cause of resistance to aminoglycosides in clinical cases of drug-resistant tuberculosis. The mechanism of aminoglycoside acetylation by *Mt*Eis is not completely understood. A systematic analysis of steady-state kinetics of acetylation of kanamycin A and neomycin B by Eis as a function of concentrations of these aminoglycosides and the acetyl donor, acetyl coenzyme A, reveals that *Mt*Eis employs a random-sequential bisubstrate mechanism of acetylation and yields the values of the kinetic parameters of this mechanism. The implications of these mechanistic properties for the design of inhibitors of Eis and other aminoglycoside acetyltransferases are discussed.

## Introduction

The emergence and spread of multidrug-resistant bacteria is a worldwide problem that requires deep understanding of the resistance mechanisms to develop novel rational approaches to antibacterial therapy. There are several mechanisms of bacterial drug resistance and one is chemical drug modification by the pathogen. *Mycobacterium tuberculosis* (*Mt*) is a notoriously pervasive infectious bacterium, whose multidrug-resistant strains are steadily spreading globally. A large fraction of clinical isolates of *M. tuberculosis* that are resistant to a second-line anti-tuberculosis antibiotic, the aminoglycoside (AG) kanamycin A (KAN), do not bear any ribosomal mutations that weaken inhibition by AGs [1], [2]. Instead, these strains harbor upregulating mutations in the promoter of the *eis* (enhanced intracellular survival) gene encoding an AG acetyltransferase, Eis [1]. KAN acetylated by *Mt*Eis does not have any antibacterial activity [1], [3]. *Mt*Eis, unlike other AG acetyltransferases, is capable of acetylating AGs efficiently at several amino groups, thus displaying an unusual regioversatility and strong AG inactivation properties [4]. Several AGs that are used in clinic and the second-line anti-tuberculosis drug capreomycin are rendered inactive by the acetylating activity of *Mt*Eis [5]. Moreover, some acetylation positions are unique to this enzyme, since they are not modified by other acetyltransferases [3]. Because Eis homologs are found in many other bacteria in addition to mycobacteria [6], [7], they pose a formidable challenge as an AG resistance factor. Moreover, because of the broad substrate versatility of Eis, a traditional approach to overcoming Eis-based resistance by designing a novel AG antibiotic that cannot be acetylated by Eis, is not likely to succeed.

The kinetic mechanism of AG acetylation by *Mt*Eis remains incompletely understood. Similarly to other acetyltransferases, *Mt*Eis uses acetyl coenzyme A (AcCoA) as the acetyl group donor and, therefore, *Mt*Eis is a bisubstrate enzyme. The AG substrate and the AcCoA can bind the enzyme either in a strict order or randomly to form the preacetylation ternary complex. While some AG acetyltransferases were demonstrated to obey a random sequential mechanism [8]–[11], others employ an ordered sequential mechanism where AcCoA needs to bind the enzyme first followed by the AG [12]–[14], and some of the enzymes follow one or the other mechanism depending on the AG scaffold [15]. A mechanism where the AG must bind the enzyme first has not been reported yet, to our knowledge. We recently demonstrated that a homolog of *Mt*Eis from *Mycobacterium smegmatis* obeys the random sequential mechanism of KAN acetylation [6]. Here, we report a systematic kinetic analysis of the clinically relevant enzyme, *Mt*Eis, with two AGs, KAN and neomycin B (NEO). KAN, a member of the 4,6-disubstituted 2-deoxystreptamine family of AGs, was selected because it is a second-line drug used in treatment of extensively drug-resistant tuberculosis. NEO was chosen as a representative of another major family of AGs, the 4,5-disubstituted 2-deoxystreptamine, in order to test if the mechanism of multiacetylation by *Mt*Eis is dependent on the molecular scaffold of the drug.

## Materials and Methods

### Materials

*Mt*Eis was expressed and purified as previously reported [4]. KAN, NEO, and AcCoA were purchased from Sigma-Aldrich (St. Louis, MO, USA) and used without any further purification.

### Steady-state acetyltransferase assays

Reactions were carried out in Tris buffer (50 mM, pH 8.0) using varying concentrations of KAN or NEO (0, 20, 50, 100, 250, 500, 1000, and 2000 µM) at several concentrations of AcCoA (25, 50, 100, 200, 300, and 500 µM) using constant concentrations of Eis (0.25 µM) and 5,5′-dithiobis-(2-nitrobenzoic acid) (DTNB, 2 mM). Reactions were monitored using a SpectraMax M5 multimode plate reader by taking absorbance measurements at 412 nm every 15 s for 15 min. Initial rates were calculated using the first 1.5 min of the reaction.

### Analysis of the bisubstrate kinetics of AG acetylation by MtEis

A random sequential rapid equilibrium bisubstrate mechanism is given by the following kinetic scheme [6]:
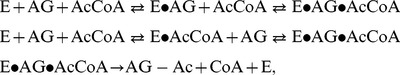
(1)where E designates the enzyme, AG and AG-Ac is the aminoglycoside and its acetylated form, respectively, and AcCoA and CoA are acetyl coenzyme A and coenzyme A, respectively. Then, under pseudo-first order conditions (large excess of the two substrates over the enzyme), the apparent Michaelis-Menten parameters in terms of the microscopic mechanism parameters are:
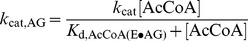
(2)

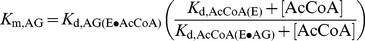
(3)

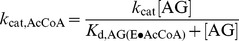
(4)

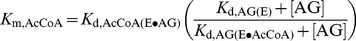
(5)Here, the subscript “AG” for *K*_cat_ and *K*_m_ means that this constant is obtained from the dependence of the steady-state rate on the concentration of AG measured at a fixed concentration of AcCoA. The analogous nomenclature is used for AcCoA. For the subscript nomenclature of equilibrium binding constants *K*_d_, for example, *K*_d,AG(E•AcCoA)_ is the equilibrium constant for binding of AG to E•AcCoA complex, etc; *k*_cat_ is the microscopic rate constant of the acetylation step (last equation in scheme (1)).

In the scheme for the ordered mechanism in which AcCoA must bind the enzyme first, the above parameters have the following functional form:
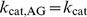
(6)

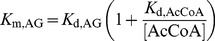
(7)

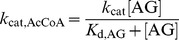
(8)

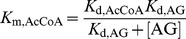
(9)The ordered mechanism is ruled out by the observed kinetics, as described in the Results and Discussion section. Because the kinetics were measured in sets of experiments at different fixed concentrations of AcCoA for each concentration of AcCoA varying the concentration of AG, a statistically rigorous way of determining the microscopic mechanism constants *K*_d_ and *k*_cat_ from the data is to determine the apparent Michaelis-Menten constants *k*_cat,AG_ and *K*_m,AG_ first. The next step is to fit dependence of *k*_cat,AG_ on [AcCoA] described by eq. (2) to the respective observed values, to obtain *k*_cat_ and *K*_d,AcCoA(E•AG)_. These values are obtained for each AG independently. Finally, because *K*_d,AcCoA(E)_ is AG-independent, its value is determined by a nonlinear regression data fitting of eq. (3) to the observed *K*_m,AG_ dependence on [AcCoA] for KAN and NEO together. In the same fitting procedure two independent values *K*_d,AG(E•AcCoA)_ for KAN and NEO are obtained. This analysis was performed by nonlinear regression with SigmaPlot 11.0 (SysStat). We have observed a preparation-dependent activity of Eis, varying within a 4-fold range. For this reason, all experiments in this study were performed with the same preparation of *Mt*Eis. The differences in fraction of active *Mt*Eis translate in corresponding differences in values of *k*_cat_ reported in this and other studies. Values of *K*_m_ and *K*_d_ as well as the relative differences in *k*_cat_ values for different AGs are not affected by this variability.

## Results and Discussion

### Steady-state kinetic measurements of KAN and NEO acetylation by MtEis

**Figure 1:**
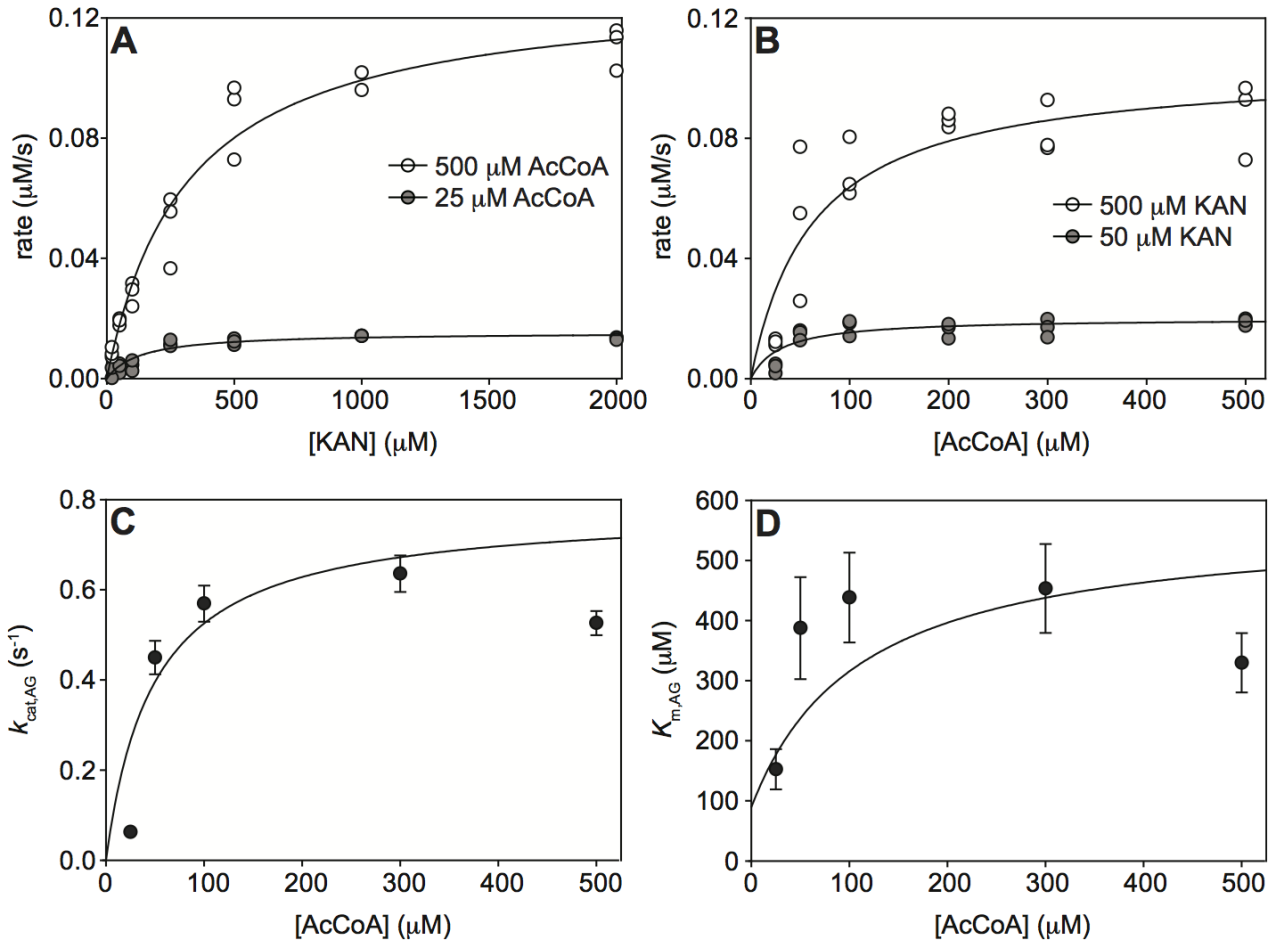
Steady-state kinetics of KAN acetylation by *Mt*Eis and their analysis. **A.** Representative dependences of the steady-state rate of acetylation of KAN on the concentration of KAN at different concentrations of AcCoA, as specified. **B.** Representative dependences of the steady-state rate of acetylation of KAN on the concentration of AcCoA at different concentrations of KAN, as specified. **C.** Dependence of the apparent rate constant (*k*_cat,AG_), as obtained from data shown in panel **A**, on the concentration of AcCoA. **D.** Dependence of the apparent *K*_m,AG_, as obtained from data shown in panel **A**, on the concentration of AcCoA. The theoretical curve in **D** is the best simultaneous fit of eq. (3) to these values and those for acetylation of NEO as described in the text.

**Figure 2:**
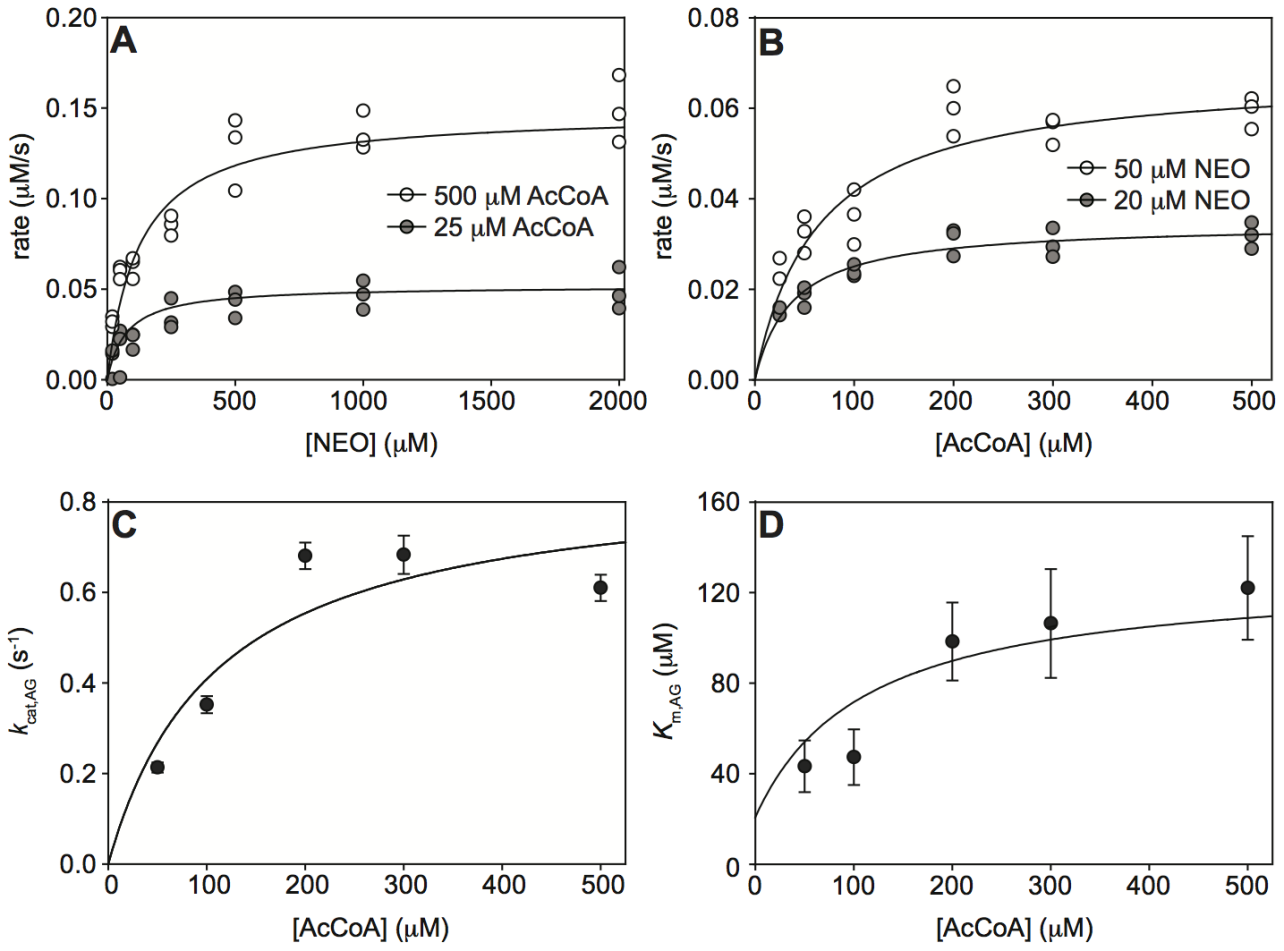
Steady-state kinetics of NEO acetylation by *Mt*Eis and their analysis. **A.** Representative dependences of the steady-state rate of acetylation of NEO on the concentration of NEO at different concentrations of AcCoA, as specified. **B.** Representative dependences of the steady-state rate of acetylation of NEO on the concentration of AcCoA at different concentrations of NEO, as specified. **C.** Dependence of the apparent rate constant (*k*_cat,AG_), as obtained from data shown in panel **A**, on the concentration of AcCoA. **D.** Dependence of the apparent *K*_m,AG_, as obtained from data shown in panel **A**, on the concentration of AcCoA. The theoretical curve in **D** is the best simultaneous fit of eq. (3) to these values and those for acetylation of KAN as described in the text.

In order to distinguish among the two ordered sequential mechanisms and a random sequential mechanism of binding of the AG and the AcCoA to *Mt*Eis to form a ternary acetylation complex, we performed a series of steady-state acetylation kinetic assays as a function of two independent variables, the concentrations of AG and AcCoA, both in large excess of the enzyme. These experiments were carried out with two AGs, KAN and NEO. For both KAN and NEO, the steady-state rate of acetylation by *Mt*Eis followed a hyperbolic dependence when plotted as a function of concentration of AG at a fixed concentration of AcCoA (Figures 1A and 2A for KAN and NEO, respectively) or as a function of concentration of AcCoA at a fixed concentration of AG (Figures 1B and 2B for KAN and NEO, respectively). Each of such hyperbolic dependences for KAN and NEO, where the concentration of AG is an independent variable (Figures 1A and 2A, respectively) yields apparent Michaelis-Menten constants, *K*_m,AG_ and *k*_cat,AG_. Dependence of these constants on the concentration of AcCoA is also hyperbolic within the experimental uncertainty (Figures 1C, 1D for KAN and Figures 2C, 2D for NEO). We observe that *K*_m,AG_ increases with increasing concentration of AcCoA (Figures 1D and 2D) for both KAN and NEO; this is possible only for a random sequential mechanism (scheme (1), eq. (3)), where *K*_d,AcCoA(E)_<*K*_d,AcCoA(E•AG)_, *i.e.*, AcCoA has a higher affinity towards free enzyme than towards AG bound enzyme. In other words, binding of AcCoA and AG is anti-cooperative. A similar observation was made recently about KAN acetylation by the Eis homolog from *M. smegmatis* based on a more limited set of experiments [6].

The rapid equilibrium mechanism of acetylation by *Mt*Eis makes physical sense, when one considers the ability of *Mt*Eis to efficiently acetylate AGs at multiple amino groups. This random sequential mechanism would allow an AG bound to *Mt*Eis to simply change its orientation in the active site after one acetylation, independently of dissociation of the CoA product and binding of another AcCoA for subsequent acetylation of the same AG. In contrast, if, for example, AG binding strictly followed AcCoA binding, the AG would need to dissociate after each acetylation event in order to rebind the same or another enzyme bound to AcCoA.

The quantitative analysis of these kinetic data in terms of the random sequential mechanism yields microscopic Michaelis-Menten parameter values for KAN and NEO. We obtain similar values of *k*_cat_ for KAN and NEO (*k*_cat_ = 0.68±0.15 s^−1^ and 0.86±0.16 s^−1^) (Figure 1A, 1C and Figure 2A, 2C) and a somewhat higher affinity of AcCoA to the KAN bound enzyme than to the NEO bound enzyme (*K*_d,AcCoA(AG•E)_ = 45±37 µM and 111±62 µM, for KAN and NEO, respectively). On the other hand, NEO binds the AcCoA bound enzyme with an approximately 3-fold higher affinity than KAN (Figures 1D and 2D); the values of the equilibrium binding constants obtained from the simultaneous fit of the KAN and NEO acetylation data (see Materials and Methods) are *K*_d,AG(AcCoA•E)_ = 439±52 µM and 135±53 µM for KAN and NEO, respectively. This analysis also yields the equilibrium constant for binding of AcCoA to *Mt*Eis, *K*_d,AcCoA(E)_ = 18±14 µM. It is to note that this value of *K*_d,AcCoA(E)_ is about 4-fold lower than that for AcCoA binding to Eis from *M. smegmatis* reported recently [6] and not equal to it, as it was assumed. Because the four equilibria in scheme (1) form a thermodynamic cycle, any three *K*_d_ values yield the fourth one, in this case the equilibrium constant for binding of AG to free enzyme, *K*_d,AG(E)_ = *K*_d,AcCoA(E)_ *K*_d,AG(AcCoA•E)_/*K*_d,AcCoA(AG•E)_ = 176±144 µM and 22±17 µM for KAN and NEO, respectively. These results demonstrate quantitatively the anti-cooperativity of AcCoA and AG binding, as discussed above based on qualitative grounds. A different, four-ring structure of NEO from the three-ring structure of KAN may explain stronger binding of NEO to *Mt*Eis. In addition, NEO is tri-acetylated by *Mt*Eis while KAN is di-acetylated [3], [4], indicating that NEO binds *Mt*Eis in more orientations than KAN does, which may explain the higher affinity of NEO to *Mt*Eis.

### Relationship to other AG acetyltransferases and considerations for the design of MtEis inhibitors

The random sequential mechanism is more common among characterized AG acetyltransferases than the ordered sequential mechanism where AcCoA binds the enzyme first. The ability of either AG or AcCoA to bind the free enzyme raises a possibility that bisubstrate compounds combining the chemical features of both substrates can be developed as *Mt*Eis inhibitors, which would be more potent than respective substrate analog inhibitors individually or as a combination. The 4,5-disubstituted 2-deoxystreptamine four-ring scaffold of NEO appears to be more promising than the 4,6-disubstituted 2-deoxystreptamine three-ring scaffold of KAN for such design, based on the stronger affinity of NEO to *Mt*Eis. Bisubstrate inhibitors of GCN5-related *N*-acetyltransferases have been reported [16]–[21]. Blanchard and colleagues developed and elegantly applied analysis of the inhibition kinetics by such inhibitors to the dissection of the kinetic mechanism, an alternative to the analysis presented in this study [22]. Consistent with the thermodynamic argument made above, a bisubstrate inhibitor of the *E. coli* AAC(3)-IV acetyltransferase, which obeys the random sequential mechanism, is extremely strong, and its intrinsic *K*_i_ could be obtained only by extrapolation [22]. These examples demonstrate the power of bisubstrate inhibitors as chemical probes. Even though therapeutically useful bisubstrate inhibitors of acetyltransferase targets have not emerged yet, examples of bisubstrate inhibitors of other enzymes that are used in clinic exist [22]. Development of a potent bisubstrate inhibitor of *Mt*Eis as a selective probe or a pharmaceutical lead, based on its unique structure and catalytic properties, is an attractive direction for future studies. Studies focusing on the development of such bisubstrate inhibitors of *Mt*Eis are currently underway in our laboratories.
